# Customized 3D orthopedic exoprostheses for dogs with amputations and congenital malformations: a case series study

**DOI:** 10.3389/fvets.2026.1854874

**Published:** 2026-06-03

**Authors:** Miriã Mamede Noronha de Souza, Ramon Rodrigues de Lima, Dannielly Virgínia de Araújo, Antonio Flávio Medeiros Dantas, Lucas Rannier Ribeiro Antonino Carvalho

**Affiliations:** 1 Postgraduate Program in Animal Science and Health, Center for Rural Health and Technology, Federal University of Campina Grande, Patos, Brazil; 2 3D Medicine Vet, João Pessoa, Brazil; 3 Veterinary Medicine Residency Program, Federal Rural University of Pernambuco (UFRPE), Recife, Brazil; 4 Department of Physiology and Pharmacology – Fyfa, Karolinska Institutet, Stockholm, Sweden

**Keywords:** canine, prosthetics, rehabilitation, socket, three-dimensional printing

## Abstract

Limb loss, particularly in cases of partial amputation, and congenital limb malformations in dogs may affect mobility, biomechanics, and quality of life. In this context, customized orthopedic exoprostheses emerge as a promising therapeutic alternative, capable of partially restoring locomotor function, redistributing loads, and improving postural stability. This study aimed to describe the development and clinical application of customized three-dimensional (3D) orthopedic exoprostheses in dogs with amputations and congenital malformations, using a case series design. Six canine patients were included, three with involvement of thoracic limbs and three with involvement of pelvic limbs. Regarding etiology, four cases were associated with partial limb amputations and two with congenital malformations. All patients underwent clinical evaluation, anatomical measurements, and individualized prosthetic planning. The three-dimensional models were developed in software and printed in polylactic acid (PLA) using fused deposition modeling (FDM) technology. After hand-finishing, padding, and securing with compressive bandages, the devices were adapted to the patients’ residual limbs. Clinical monitoring was conducted, with emphasis on assessing functionality, skin integrity, gait stability, and response to rehabilitation. The results demonstrated good acceptance by the animals, with no skin lesions, mechanical failures, or definitive refusal to use the devices. In all cases, improvements in mobility, stability, and quality of life were observed, with progressive adaptation and functional return to locomotion. Therefore, this study highlights the clinical potential of producing veterinary orthopedic exoprostheses using 3D printing, emphasizing its potential as an accessible, personalized, and functional strategy for the rehabilitation of dogs with missing or malformed limbs.

## Introduction

Exoprostheses are devices that function as partial or total substitutes for lost limbs, contributing to body weight redistribution and assisting in postural stability and locomotor functionality during stance and ambulation (1, 2). In animals, limb loss and the resulting biomechanical alterations directly impact locomotor performance and overall well-being and are associated with reduced social activity (3, 4).

Orthopedic disorders affect a relevant proportion of the animal population and are frequently associated with traumatic injuries (5). Complete limb amputation is widely performed in veterinary medicine and has been associated with generally favorable outcomes, with most dogs achieving good functional adaptation and satisfactorily to good quality of life following surgery (6, 7). Similar outcomes have also been reported in feline populations (8). However, variations in activity levels, exercise tolerance, and behavioral patterns have been reported in a subset of cases, as perceived by owners (6, 7).

In contrast, partial limb amputation remains less frequently reported in the literature, with limited evidence regarding long-term functional outcomes and owner-reported satisfaction. Although functional adaptation may occur, partial limb loss has been associated with a higher rate of complications and less well-defined long-term outcomes, highlighting a gap in current knowledge (9).

These conditions, although etiologically distinct, share a common outcome characterized by limb deficiency and consequent biomechanical imbalance. Another cause of biomechanical alteration is congenital abnormalities which represent one of the main causes of neonatal disorders and are characterized by structural and/or functional deformities that develop during gestation or are present at birth, potentially compromising the animal’s viability (10).

Similar to amputations, which may result from both conditions, partial or total limb loss can affect the animal’s quality of life, hindering participation in rehabilitation therapies and increasing the risk of secondary deformities and joint degeneration, in addition to reducing physical activity levels and functional autonomy (11).

In this context, additive manufacturing technology has emerged as a transformative tool in veterinary medicine, with applications across different fields, including orthopedics, dentistry, and, more recently, surgical planning. According to Lima and Carvalho (12), this technology enables the creation of anatomical models from imaging examinations, facilitating structural visualization, and supporting clinical decision-making. Furthermore, the availability of affordable printers and software has increased the feasibility of this approach.

In veterinary medicine, orthotic and prosthetic devices are used to support locomotor function in patients with limb loss or impairment. Prostheses can be classified as endoprostheses, when surgically anchored to bone structures, or exoprostheses, when externally fitted to the residual limb, with the primary aim of restoring stability and reducing the biomechanical alterations associated with segmental absence (11). Orthoses, in turn, are external devices designed to support, protect, or control the movement of body segments, allowing restriction or guidance of joint mobility according to clinical needs (13).

In addition, prostheses and orthoses produced through 3D printing have become increasingly important for the functional reintegration of patients, promoting restoration of well-being and segmental kinetic recovery, as well as reducing pain associated with prolonged biomechanical overload (4, 14). Within this framework, different thermoplastic materials can be employed, including polylactic acid (PLA), thermoplastic polyurethane (TPU), and nylon, each with distinct mechanical properties that directly influence device performance under weight-bearing conditions. While PLA is widely used due to its ease of processing and biocompatibility, it presents limited impact resistance (15), TPU provides superior flexibility and energy absorption, which are advantageous in dynamic loading environments (16), whereas polyamides, such as nylon, offer higher mechanical strength and durability, making them more suitable for high-stress applications in locomotor devices (17).

The effective design and fabrication of orthopedic exoprostheses require a comprehensive understanding of anatomical, kinetic, and biomechanical principles to ensure optimization of the interaction between the device and the patient (18). The suitability of animals for exoprosthetic rehabilitation is influenced by multiple factors, including species, patient temperament and behavior, neurological and orthopedic status, as well as the caregiver’s ability to maintain appropriate follow-up and daily management of the device (19). In addition, the use of these devices may be discouraged in aggressive animals because of the difficulties associated with handling, adaptation, and long-term safe use (13). Regarding residual limb characteristics, preservation of approximately 50% of the radius/ulna or tibia/fibula has been associated with improved prosthetic attachment and may facilitate the use of socket-based prostheses when clinically indicated for rehabilitation (20).

Despite technological advances, there remains a scarcity of literature addressing the clinical application of 3D-printed orthopedic exoprostheses in dogs, particularly considering the anatomical, adaptive, and biomechanical challenges associated with these devices. Considering this scenario, the present study aimed to describe the development and application of customized 3D-printed orthopedic exoprostheses in dogs with partial limb amputations and congenital malformations affecting limb development, through a case series using three-dimensional modeling and 3D printing techniques as a functional and accessible therapeutic alternative.

## Methods

This study was conducted in collaboration with the private company 3D Medicine—3D Solutions for Health, located in João Pessoa, Brazil. The study was performed in accordance with ethical principles and approved by the Animal Use Ethics Committee of the Federal University of Campina Grande (CEUA/CSTR) under protocol number 44.2024.

The development of digital models for the fabrication of customized exoprostheses was performed using the three-dimensional computer-aided design (CAD) software Blender^®^ (Blender Foundation, Amsterdam, Netherlands), based on direct anatomical measurements of each patient obtained using measuring tape, digital calipers, and rulers. Advanced imaging techniques, such as computed tomography or three-dimensional rendering, were not used. The main collected parameters included residual limb height relative to the ground with proximal joints anatomically aligned, residual limb circumference at three different levels, limb diameter, and the distance between the distal end of the residual limb and the nearest proximal joint. These measurements were used to create a simple cylindrical three-dimensional digital phantom of the residual limb, intended primarily to provide a spatial reference for the anatomical dimensions and proportions of each patient. This phantom served as the anatomical basis for individualized prosthetic modeling, allowing adaptation of socket dimensions, limb alignment, and prosthetic height according to the morphology and biomechanical requirements of each animal.

The three-dimensional files were exported to the slicing software BambuStudio^®^ (v.1.10.1.50, BambuLab, Shenzhen, China), which generated layer-by-layer printing instructions for Fused Deposition Modeling (FDM) technology. The devices were manufactured using an X1 Carbon Combo 3D printer (BambuLab, Shenzhen, China), with polylactic acid (PLA) selected as the printing material due to its biocompatibility, compressive strength, low cost, and renewable origin (derived from corn starch and sugarcane) (21).

The printing protocol was adapted from Carvalho (16) and included the following technical parameters: 0.4-mm nozzle diameter, 0.2-mm layer height, 1.6-mm wall thickness, 50% gyroid infill pattern, printing speed ranging from 200 to 250 mm/s, and tree-type support structures when required. All prostheses were developed using standardized technical parameters, including material selection and printing configurations, to ensure adequate mechanical resistance, reduced weight, and patient comfort.

After printing, support structures were manually removed, and each component underwent fine sanding for surface finishing. Subsequently, the exoprostheses were internally lined with acrylic padding and cushioned fabric to enhance comfort, prevent abrasions, and improve adaptation to the residual limb. Fixation was achieved using compressive bandages individually adjusted according to the morphology of the residual limb. Additionally, the bases of the prostheses were fitted with anti-slip rubber soles to improve ground traction and prevent slipping during ambulation.

Immediately after prosthesis fitting, device acceptance was qualitatively evaluated based on weight-bearing capacity, gait performance, and behavioral response to the device. Acceptance was classified as: (1) poor, characterized by hesitation or resistance to prosthesis use, inconsistent or absent weight-bearing, and difficulty initiating ambulation; (2) satisfactory, characterized by partial functional adaptation, progressive weight-bearing, and gradual gait improvement during the adaptation period; and (3) good to excellent, characterized by immediate spontaneous use of the prosthesis, consistent weight-bearing from the first fitting, stable ambulation, and absence of evident discomfort or resistance to the device.

Clinical follow-up included periodic assessments of functional adaptation, skin integrity, gait stability, and overall tolerance to the prosthetic device. Professional physiotherapeutic follow-up was recommended for all patients; however, owner adherence to specialized rehabilitation programs was limited. Therefore, only general home-based rehabilitation guidance was provided to the owners, consisting of controlled leash-guided walking, assisted weight-shifting exercises, gradual stimulation of supported stance, and proprioceptive stimulation on different floor surfaces. Exercise intensity and progression were empirically adjusted according to each patient’s tolerance and clinical adaptation, following general principles of veterinary rehabilitation (22).

## Case reports

All dogs included in this study presented with amputated or malformed residual limbs affecting either thoracic or pelvic limbs and underwent adaptation to customized exoprostheses manufactured through 3D printing. Table 1 summarizes the clinical characteristics and the underlying conditions that indicated the use of these devices, while Table 2 presents data related to residual limb measurements, number of prosthetic adjustments, immediate device acceptance, and follow-up duration.

**Table 1:** Clinical characterization of dogs fitted with customized exoprostheses manufactured using 3D printing.

Case	Breed	Sex	Age	Weight (kg)	Affected limb	Level of articular involvement	Clinical history
1	Mixed-breed	M	3 years	17.5	Right pelvic limb	Tarsal joint	Congenital malformation
2	Mixed-breed	M	8 years	13.3	Left pelvic limb	Tarsal joint	Traumatic amputation
3	Labrador Retriever	M	10 years	40	Left thoracic limb	Carpal joint	Surgical amputation
4	Mixed-breed	M	13 years	14	Right thoracic limb	Carpal joint	Traumatic amputation
5	Mixed-breed	M	3 years	15.3	Left thoracic limb	Proximal radioulnar third	Traumatic amputation
6	Mixed-breed	F	9 months	11.6	Left pelvic limb	Interphalangeal joint	Congenital malformation

The table summarizes the main clinical characteristics of dogs fitted with customized 3D-printed exoprostheses, including breed, sex, age, body weight, affected limb, level of articular involvement, and the underlying etiology of amputation or malformation.

**Table 2:** Residual limb characteristics, prosthetic adaptation, and follow-up information of dogs fitted with customized 3D-printed exoprostheses.

Case	Stump length (cm)	Stump circumference (cm)	Distance from stump to ground (cm)	Number of prosthetic adjustments	Immediate device acceptance	Follow-up duration (months)
1	11.5	15.7	8	0	Satisfactory	4
2	13	12.6	15	0	Poor	4
3	15	12	6	0	Good/excellent	4
4	13	12.6	10	0	Good/excellent	3
5	15	11	20	2	Poor	8
6	6	9	8	1	Satisfactory	9

The clinical reports of dogs undergoing adaptation to customized 3D-printed exoprostheses are presented below, with emphasis on the characteristics of the affected limb, fabrication process, adaptation period, and response to the device.

**Figure 1:**
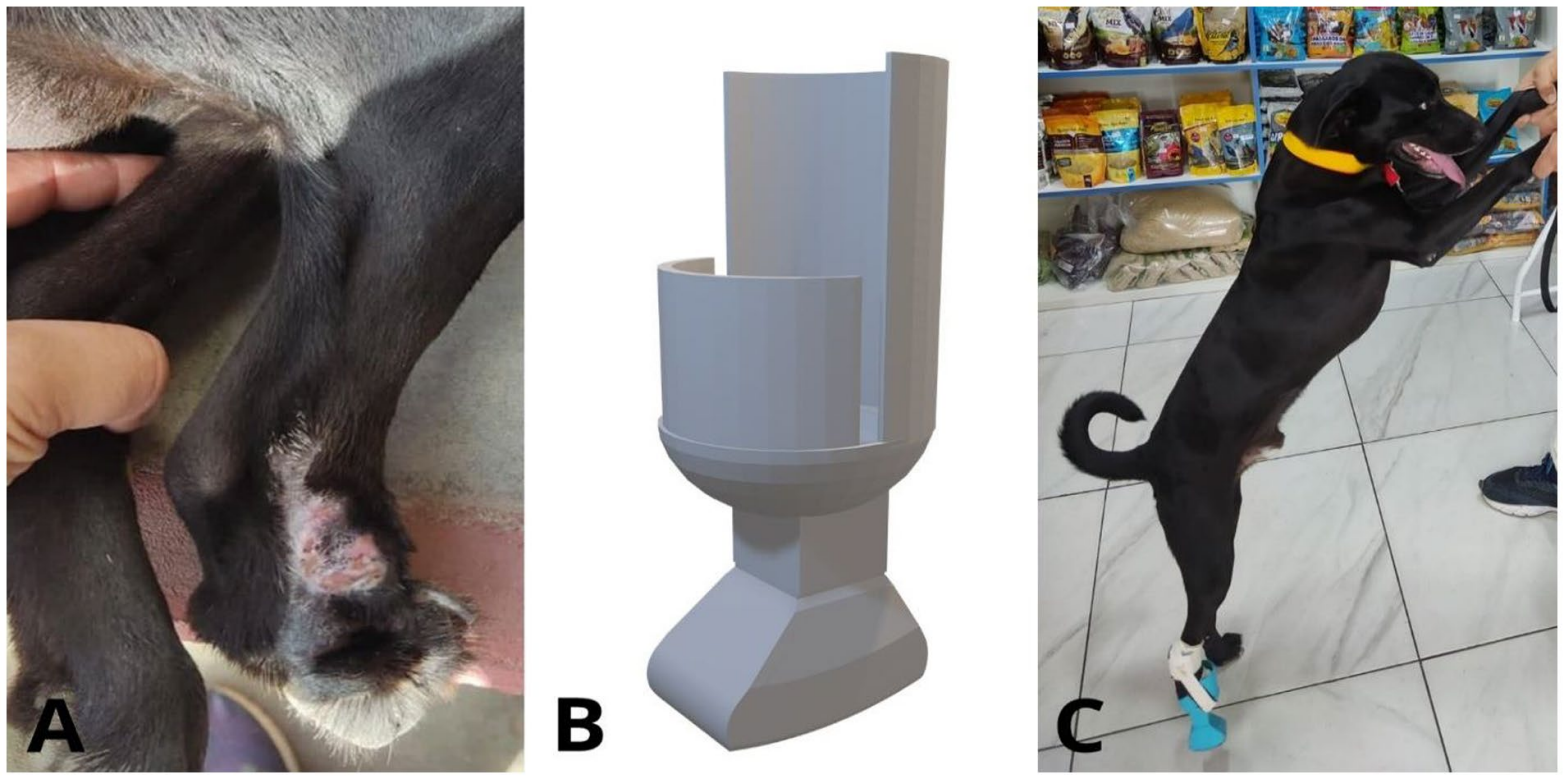
**(A)** Lateral view photographic record of the congenital malformation in the right pelvic limb of a male dog. **(B)** Customized virtual model of the 3D socket-style exoprosthesis. **(C)** Immediate record after the first fixation of the 3D prosthesis, showing the support of the amputated limb on the structure, with balanced distribution of body weight on two bases, offering greater stability to the animal.

*Case 1*. A 3-year-old, 17.5-kg male mixed-breed dog presented with a history of congenital malformation affecting the right pelvic limb, involving the tarsal joint. The condition resulted in significant locomotor impairment, absence of proprioception, lameness, and skin lesions caused by direct contact of the malformed structure with the ground (Figure 1A). Given these limitations, the fabrication of a customized 3D-printed exoprosthesis was indicated as an alternative to improve functionality and overall quality of life. Detailed measurements of the affected limb were collected and used to develop a three-dimensional model, which was subsequently manufactured using 3D printing (Figure 1B). The measurements collected were stump distance to the ground of 8 cm, residual limb length of 11.5 cm, and circumference of 15.7 cm. From the first exposure to the exoprosthesis, the dog demonstrated satisfactory adaptation, with partial weight-bearing on the affected limb and more balanced body weight redistribution. The owner actively participated in the rehabilitation process, carefully following the instructions provided by the veterinarian and technical team (Figure 1C). Short-term clinical follow-up confirmed complete adaptation to the prosthetic device. No skin lesions, discomfort, or complications related to prosthesis use were observed. The successful adaptation highlighted the effectiveness of the device and the improvement in the animal’s quality of life, allowing stable and functional locomotion over time.

**Figure 2:**
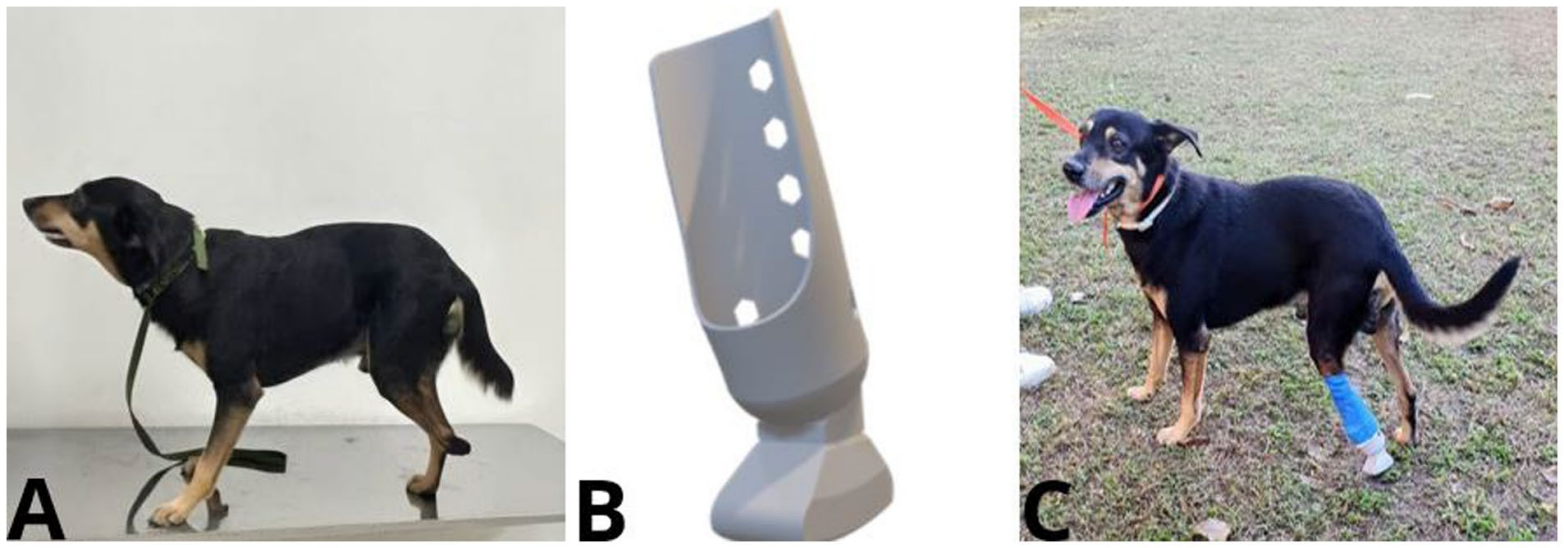
**(A)** Lateral view photographic record of a dog with a history of traumatic amputation of the left pelvic limb, resulting from a stab wound. **(B)** Virtual model of the angled 3D exoprosthesis, made from precise measurements of the residual stump, designed with a wider base to promote stability. **(C)** First fixation of the device to the stump, showing support of the prosthetic limb and the beginning of functional adaptation to gait.

*Case 2*. An 8-year-old male mixed-breed dog weighing 13.3 kg was presented with a history of traumatic amputation of the left pelvic limb at the distal tarsal level. The amputation resulted from a stab wound, constituting an act of animal abuse that occurred outdoors, severely compromising the animal’s tarsal joint. After the initial period of complete healing of the residual stump, the animal was referred to for specialized evaluation with the aim of creating a customized prosthesis that could restore locomotor function and improve quality of life (Figure 2A). During the initial consultation, the patient showed evident discomfort during movement, as well as increased sensitivity to palpation in the amputated region, supporting the need for an adaptive intervention. In this context, an exoprosthetic approach was considered as a therapeutic option and selected following discussion with the owner, considering the clinical condition and the goal of limb preservation. For the development of the prosthesis, detailed anatomical measurements of the stump were taken, including the distance from the stump to the ground (15 cm), the length of the limb to the stump (13 cm), and the circumference of the region (12.6 cm). This information was fundamental for the elaboration of the personalized three-dimensional model, which would allow the manufacture of an anatomical and functional device, compatible with the patient’s specificities (Figure 2B).

After the manufacture and initial adjustment of the prosthesis, the animal was fitted with the device and subjected to a progressive adaptation protocol (Figure 2C). Although it showed some hesitation and caution in the first moments of use, the dog demonstrated significant improvement in locomotion over time, demonstrating the ability to adapt to the new artificial limb. Clinical follow-up was rigorous, with constant monitoring of the functional response and the performance of necessary technical adjustments to ensure proper alignment, device stability, and comfort during use.

**Figure 3:**
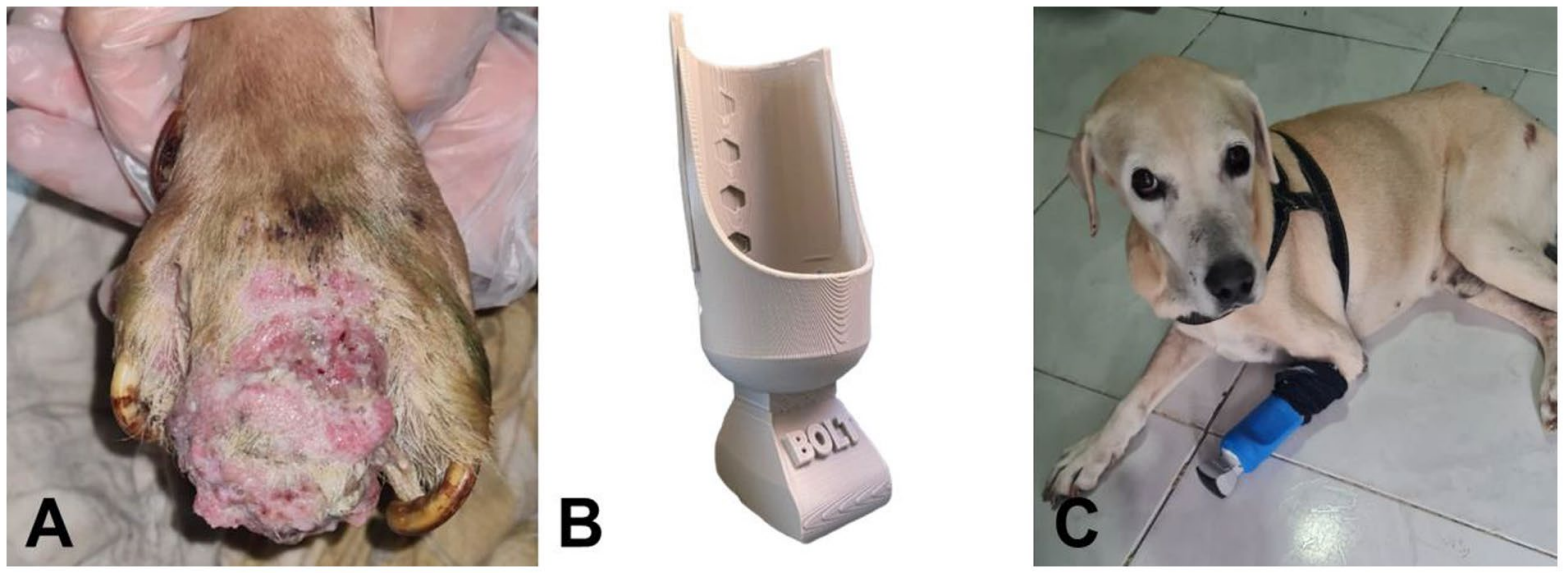
**(A)** Photographic record of the left thoracic limb before amputation due to squamous cell carcinoma. **(B)** Three-dimensional model of the 3D exoprosthesis, manufactured with reduced height and anatomical design for close adjustment to the carpus. **(C)** Dog in sternal recumbency position during the initial adaptation phase, already using the prosthesis on the left thoracic limb and demonstrating contact of the prosthetic stump with the ground.

*Case 3*. A 10-year-old neutered male Labrador Retriever was presented with a history of low amputation of the left thoracic limb, performed at the carpal level as part of treatment for squamous cell carcinoma (SCC) (Figure 3A). After initial recovery, the animal began to show signs of apathy, muscle atrophy in the amputated limb, lameness, and limited movement, factors that motivated the indication for the use of a customized exoprosthesis. During the physical evaluation, precise measurements of the stump were collected, including a length of 15 cm, a circumference of 12 cm, and a distance from the stump to the ground of 6 cm, which served for the development of the three-dimensional digital model. The model had subsequently materialized through 3D printing. The prosthesis was carefully adjusted to the limb to provide comfort and stability (Figure 3B). From the first contact with the prosthesis (Figure 3C), the dog was able to support the limb on the ground, distributing body weight evenly and significantly reducing impacts on the contralateral limb. These improvements were maintained during short-term follow-up, with benefits in mobility and comfort during locomotion.

**Figure 4:**
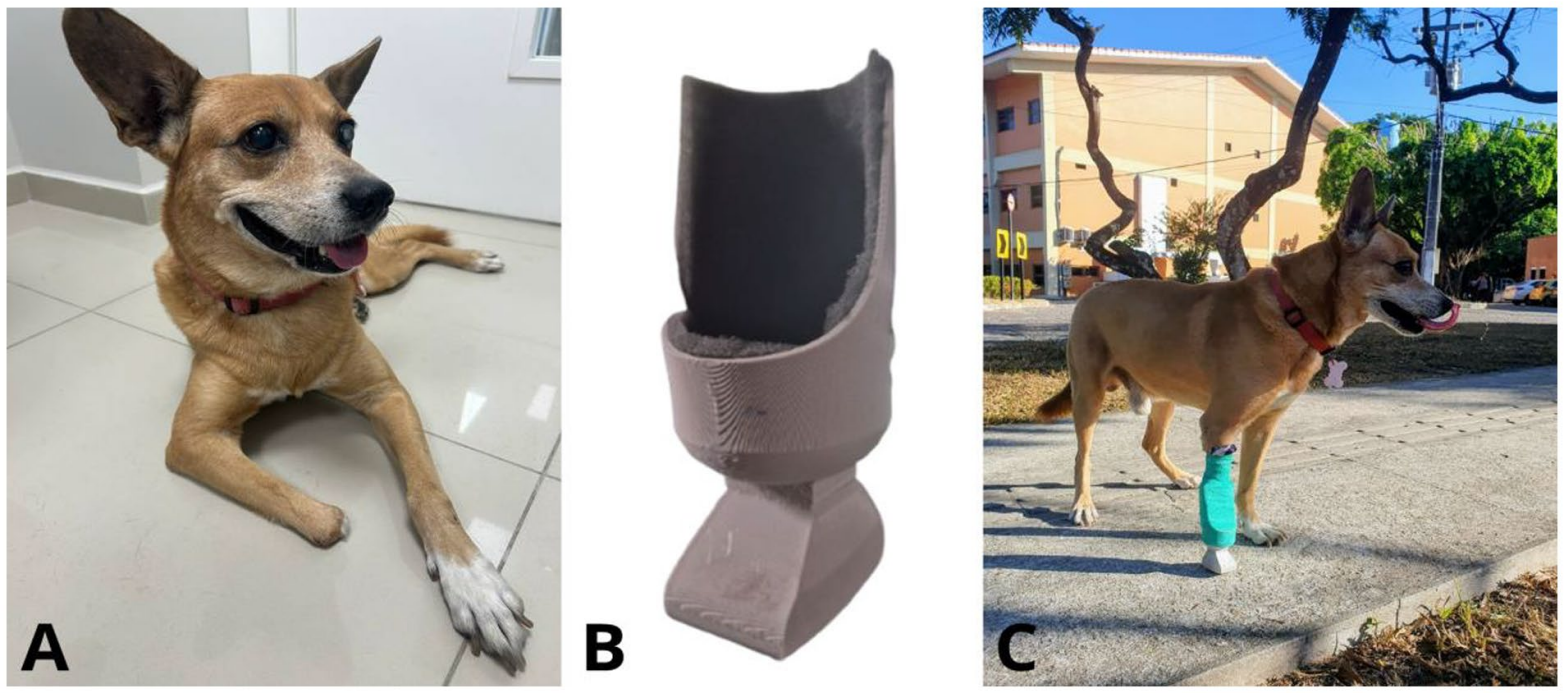
**(A)** Photographic record of a dog showing healed traumatic amputation of the right thoracic limb caused by a domestic accident. **(B)** Customized 3D exoprosthesis covered with acrylic blanket. **(C)** Record of the use of the device after adaptation, showing firm support on the ground and improvement in locomotor functionality.

*Case 4*. A 13-year-old, 14 kg, male mixed-breed dog was presented with a history of traumatic amputation of the right thoracic limb at the carpal joint. The amputation was caused by a domestic accident in which the gate of the residence trapped the limb, leading to its traumatic removal. During the initial assessment, the animal presented with a lack of proprioception and muscle atrophy in the affected limb but did not present with weight-bearing wounds or crepitus (Figure 4A). It was observed that the dog supported the stump on the ground as a compensatory mechanism during locomotion. Given these conditions, a customized exoprosthesis was developed to improve its mobility and quality of life. The measurements collected were stump distance to the ground of 10 cm, residual limb length of 13 cm, and circumference of 12.6 cm. The prosthesis was internally lined with padded material to ensure comfort and fitted to the stump with a compressive bandage, providing greater stability (Figure 4B). From the first fitting, the animal was able to successfully support the prosthetic limb on the ground, demonstrating improved functionality during gait (Figure 4C). In addition, rehabilitation guidance was provided to facilitate adaptation to the prosthetic device, including controlled leash-guided walking, assisted weight-shifting exercises, stimulation of supported stance, and proprioceptive exercises performed on different floor surfaces. Initially, the dog performed supervised walking sessions of approximately 5 min, with gradual progression according to its tolerance and adaptation. During follow-up, the owner reported improved acceptance of physical activity, reduced fatigue during ambulation, and a significant improvement in posture while feeding. Periodic short-term follow-up assessments were conducted to monitor comfort, prosthesis adaptation, locomotor performance, and overall quality of life.

**Figure 5:**
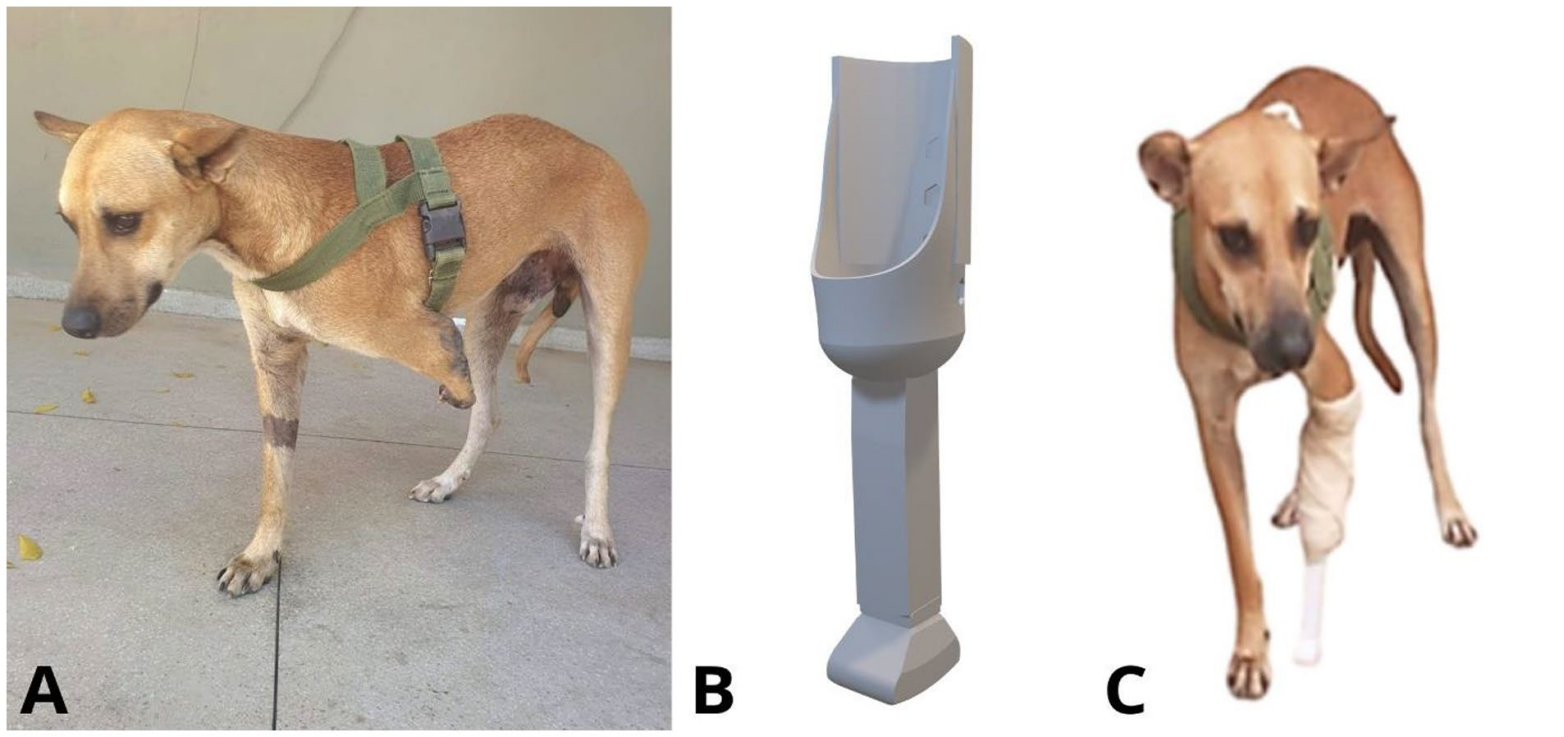
**(A)** Lateral view photograph of a dog with traumatic amputation of the left thoracic limb due to being run over. **(B)** 3D digital model of the exoprosthesis, made with an elongated height to compensate for the articular lateralization observed in the patient. **(C)** First adaptation of the prosthesis, fixed to the stump with a compressive bandage and soft inner lining, demonstrating the beginning of gait with functional support.

*Case 5*. A 3-year-old, 15.3 kg, male mixed-breed dog was presented with a history of traumatic amputation of the left thoracic limb in the proximal radioulnar third (Figure 5A). The accident occurred when the animal was only 2 months old, after a vehicle ran over the limb, resulting in its partial loss of the limb. At the time, the dog was taken to the veterinary hospital, where it received emergency care and treatment for the open wound until complete healing. During the process, an infection occurred at the site, but the condition was successfully controlled. However, the lack of physiotherapy compromised the animal’s mobility, leading to lateral deviation of the elbow joint, marked muscle atrophy of the limb, and inability to bear weight. In addition, the animal exhibited pain-related hypersensitivity during palpation of the affected limb, without evidence of active skin lesions at the time of evaluation. A custom-made exoprosthesis was developed with the aim of improving the patient’s functional capacity and quality of life, with a length of 15 cm, a circumference of 11 cm, and a distance from the stump to the ground of 20 cm (Figure 5B). The exoprosthesis was covered with acrylic batting to ensure comfort and prevent injuries or abrasions. Fixation was performed with crepe bandage, well-adjusted with the aid of adhesive tape, providing stability and safety in the use of the device.

Initially, the dog had difficulty adapting to the prosthesis, showing sensitivity in the affected region, marked limb lateralization, reduced weight-bearing capacity, and instability during stance and ambulation. Due to the greater prosthetic height and the severe biomechanical alterations observed in this case, the initial rehabilitation approach focused primarily on supported stance, gradual weight redistribution, and tolerance to prosthesis use. Early adaptation exercises included short periods of assisted standing, controlled static weight-bearing, postural support exercises, and brief leash-guided walking sessions over short distances to progressively stimulate limb loading while minimizing discomfort and fatigue. This case represented one of the most biomechanically complex scenario in the series due to marked articular lateralization, muscle atrophy, and prolonged absence of prior physiotherapy, requiring specific design adaptations to ensure stability and comfort. During follow-up, the animal demonstrated progressive improvement in locomotion and functional adaptation to the prosthesis (Figure 5C). Simultaneously, the dog was also undergoing treatment for a transmissible venereal tumor (TVT), achieving complete remission without alterations in physiological parameters.

**Figure 6:**
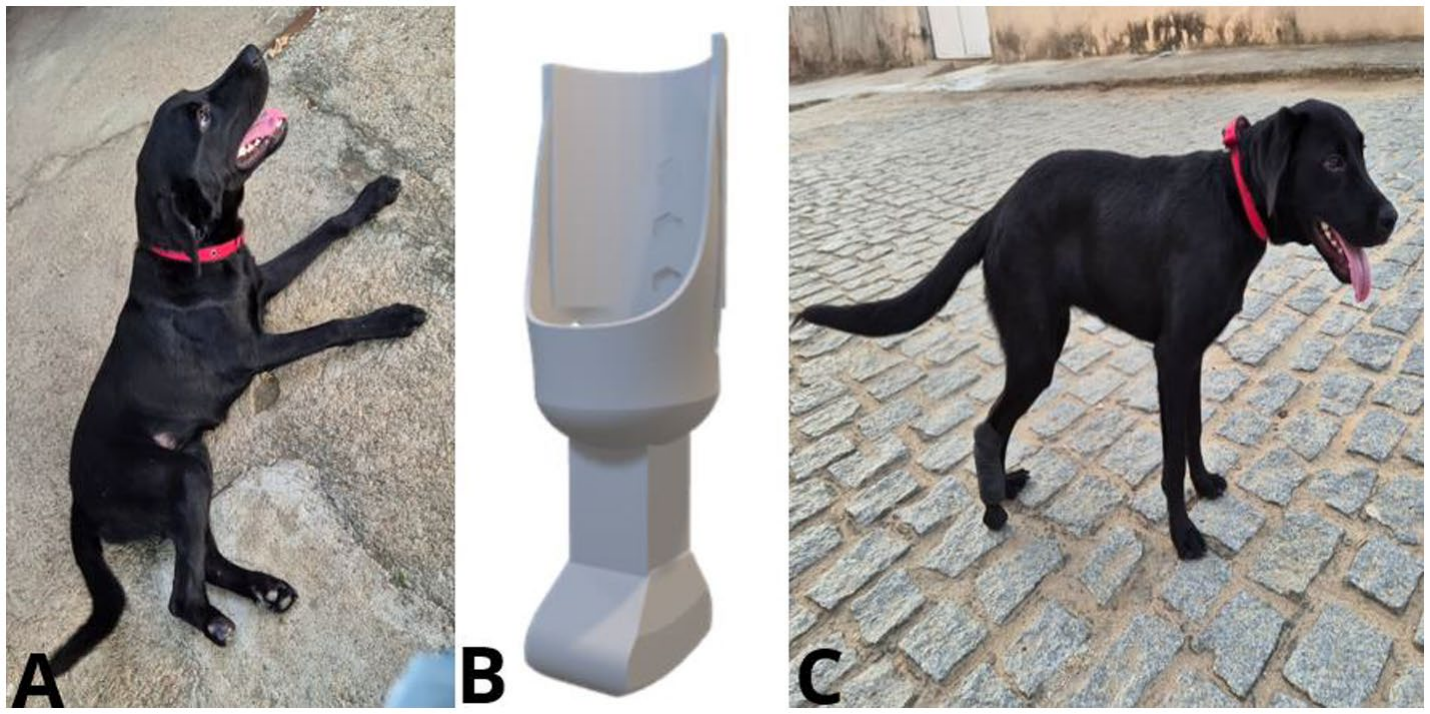
**(A)** Lateral view photograph of a female dog with a congenital malformation in the left pelvic limb, characterized by alterations in the tarsal joint. **(B)** Three-dimensional model of the 3D exoprosthesis, developed at an intermediate height and with structural reinforcement for stability. **(C)** Use of the device during the adaptation phase, showing active support of the prosthetic limb on the ground and gradual resumption of locomotion.

*Case 6*. A nine-month-old, mixed-breed female dog weighing 11.6 kg was presented with a congenital malformation of the phalanges of the left pelvic limb, distal to the tarsal joint, while maintaining limb functionality (Figure 6A). The structural anomaly involved an atypical bone conformation, characterized by the presence of two phalanges in the affected limb, preventing adequate locomotion and severely compromising limb functionality. The initial clinical evaluation included detailed visual inspection, orthopedic and neurological examination, and precise measurement of the stump for planning a customized prosthesis. The measurements collected were stump distance to the ground of 8 cm, residual limb length of 6 cm, and circumference of 9 cm. These data were fundamental for the development of a precise and individualized three-dimensional model that considered the patient’s anatomical particularities (Figure 6B). After manufacturing, the device underwent finishing, internal padding, and individualized adaptation procedures to provide stability and prevent discomfort or skin lesions at the stump interface. During the adaptation period, the patient demonstrated a progressive return to locomotion with excellent acceptance of the exoprosthesis. Initial rehabilitation guidance included supervised walking sessions lasting approximately 20 to 30 min, emphasizing gradual gait retraining, supported weight-bearing, and functional muscle conditioning. Over the course of short-term follow-up, marked improvement in mobility, balance, and locomotor confidence was observed, allowing the animal to ambulate with greater autonomy and stability. According to owner reports and clinical observations, the patient eventually regained the ability to perform more dynamic movements, including running while using the prosthetic device, resulting in a substantial positive impact on quality of life (Figure 6C).

## Discussion and conclusions

The use of three-dimensional modeling and FDM printing technologies has proven to be effective and accessible, allowing the production of customized devices according to the morphology of each patient. The anatomical variability among canine patients, although a strong point for 3D printing that allows customization, also means that each prosthesis is unique and requires a meticulous and individualized design and manufacturing process (23). In addition to bone particularities, the condition of the residual musculature proved to be a determining factor since different degrees of atrophy and muscle asymmetry influence load distribution and stability throughout the adaptation process (13, 24).

The customization enabled by 3D printing is a determining factor for increasing the functional effectiveness of prostheses, directly reflecting on the adaptation process, and improving the quality of life of animals. This degree of individualization favors faster rehabilitation processes and enhances the return of patients to their usual behavioral and locomotor functions (2).

Similarly, previous studies have demonstrated that dogs fitted with socket prostheses can achieve good functional outcomes and maintain a satisfactory quality of life, although successful adaptation is closely related to proper fit and careful management of potential complications such as pressure sores (25). In the present study, in addition to structural customization, adaptation was favored by the padded inner lining and the protection of the stump with a cotton sock, which contributed to adequate tolerance to the device, without the occurrence of clinically relevant skin complications.

The fabrication and adaptation of custom-printed orthopedic exoprostheses using 3D printing demonstrated promising results during the short-term follow-up period, with observed improvements in mobility, stability, and quality of life of the dogs treated. These findings are in line with the reports of Marcellin-Little (1) and Cruz et al. (14), who highlight the importance of prostheses in restoring locomotor functionality and preventing compensatory overload in healthy limbs.

Studies with exoprostheses reveal that, despite the animal’s initial tolerance, it requires physiotherapy follow-up and adjustments to help with adaptation over time. Models with external fixation and absence of bone fixation have less control of movements, greater mechanical wear, and worse impact absorption, especially in running activities or abrupt changes of direction, which compromises functionality and acceptance by the animal (11, 23).

Due to their snap-fit nature, they predominantly rely on external fixation methods, such as bandages, which can compromise the stability and safety of the device during use. The effectiveness of fixation is even more critical in amputations of higher limbs, where the contact area for external fixation is reduced, making it difficult to obtain a firm and lasting fit (4). In Case 5, characterized by amputation in the proximal radioulnar third, slower adaptation and initial challenges in stabilizing the device were observed, mainly due to marked muscle atrophy, articular lateralization, and pain-related hypersensitivity during limb manipulation, highlighting how more proximal levels of amputation can compromise prosthetic stability and prolong the time required for functional consolidation.

Solutions such as adaptable straps and silicone coatings have been tested, but they still do not offer ideal adhesion in dynamic movements. Thus, external fixation appears as one of the main limitations related to this type of prosthesis for animals, unlike humans where models with external fixation are widely used (26–28).

Additional engineering strategies achievable through additive manufacturing have been described to improve prosthetic stability and biomechanical performance in dogs, including anatomically contoured socket interfaces, customized load-distribution structures, and geometric modifications designed to better withstand gait forces and improve weight redistribution during locomotion (29, 30). Furthermore, finite element analysis (FEA) has been reported as a useful tool for evaluating stress distribution and mechanical resistance in 3D-printed prosthetic components subjected to gait-related loading conditions (29). Although these approaches were not evaluated in the present study, they represent relevant engineering strategies that may contribute to the optimization of veterinary exoprosthetic devices.

Regarding the rehabilitation and adaptation process, the dogs included in the present study demonstrated gradual and individualized functional responses to prosthesis use, although most owners reported noticeable improvements in mobility and overall quality of life, consistent with previous reports in dogs fitted with orthopedic prosthetic devices (13). The adaptation period varied among patients and appeared to be influenced by factors such as stump morphology, degree of muscle atrophy, biomechanical alterations, and previous locomotor compensation patterns. Similar challenges have been reported in the literature. In a prospective study involving 43 dogs fitted with orthoses or prostheses, Rosen et al. (31) observed that 91% of the animals developed at least one complication within a 12-month period, with skin-related lesions, including abrasions, hair loss, and wounds, representing the most common adverse findings.

Mechanical problems with the device (e.g., need for repair) were also described, occurring in approximately 7–40% of dogs, depending on the type of orthosis/prosthesis; non-acceptance of the device (resistance or refusal to use) in approximately 10–55%; abandonment or premature discontinuation of use before veterinary accommodation in 10–27%; and another category, such as “major” skin complications (wounds requiring veterinary treatment) versus minor ones, present in about 17% of cases among those who had skin complications (31). Comparable challenges have also been described in dogs undergoing partial limb amputation managed with socket prostheses, including variability in adaptation and the occurrence of complications (9, 25).

In all evaluated cases, favorable clinical evolution was observed, with no records of skin lesions or major complications associated with prosthesis use, suggesting that the internal coating and padding strategies adopted in the exoprostheses were effective in protecting the residual limb and minimizing discomfort during ambulation, similar to that described by Carr et al. (32), who emphasized that adjustments in internal padding thickness represent a key strategy for correcting prosthetic misalignment or limb length discrepancies and maintaining appropriate gait patterns.

Despite the favorable outcomes observed, some limitations of the present study should be considered. Radiographic evaluation was not incorporated into the prosthetic planning process, although imaging techniques could provide additional anatomical and biomechanical information useful for individualized prosthetic design, as reported by Wendland et al. (33). In addition, standardized professional physiotherapeutic follow-up was not achieved due to limited owner adherence, resulting in reliance on non-standardized home-based rehabilitation guidance, which may have influenced functional adaptation among patients, as rehabilitation is considered essential for optimizing the functional use of orthotic and prosthetic devices (18).

Another limitation was the absence of long-term follow-up, restricting the assessment of chronic biomechanical adaptations, prosthesis durability, and potential late orthopedic complications. Furthermore, objective functional assessment methods, such as kinetic or kinematic gait analysis and force platform evaluation, were not performed. Consequently, functional outcomes were based on qualitative clinical assessment of gait, weight-bearing, and prosthesis adaptation. Future studies involving larger populations, prolonged follow-up, imaging support, standardized rehabilitation protocols, and objective gait analysis are necessary to further validate the clinical applicability of customized veterinary exoprostheses.

Even considering these limitations, customized three-dimensional exoprostheses demonstrated important clinical applicability as functional and relatively low-cost rehabilitation alternatives, particularly in cases where preservation of the residual limb allowed individualized prosthetic adaptation. The accessibility of additive manufacturing technologies, combined with individualized digital modeling, may substantially expand therapeutic possibilities for veterinary locomotor rehabilitation.

In summary, three-dimensional modeling and 3D printing proved to be effective tools for the production of customized orthopedic exoprostheses in dogs, promoting improvements in mobility, postural stability, and overall quality of life. The application of additive manufacturing in the development of veterinary orthopedic exoprostheses represents an important advancement in Veterinary Medicine and highlights the potential of personalized rehabilitation strategies for animals with amputations or congenital limb deformities.

## Data Availability

The raw data supporting the conclusions of this article will be made available by the authors, without undue reservation.
